# Atrial Fibrillation Occurring After Smoking Marijuana: A Case Report and Review of the Literature

**DOI:** 10.5811/cpcem.7227

**Published:** 2024-07-11

**Authors:** Mary Unanyan, Christopher Colbert, Wesley Eilbert

**Affiliations:** University of Illinois at Chicago, College of Medicine, Department of Emergency Medicine, Chicago, Illinois

**Keywords:** *atrial fibrillation*, *marijuana-induced atrial fibrillation*, *marijuana-induced arrhythmia*, *cannabis-induced arrhythmia*, *case report*

## Abstract

**Introduction:**

Atrial fibrillation (AF) is the most common cardiac arrhythmia, occurring primarily in individuals with known risk factors such as advanced age, heart failure, and coronary artery disease. Cannabis use produces several cardiovascular changes resulting in proarrhythmic effects on the heart.

**Case Report:**

A 38-year-old woman with no significant past medical history presented to the emergency department (ED) complaining of palpitations with associated shortness of breath occurring after smoking marijuana. She was found to be in AF. Evaluation in the ED and during hospitalization found no cardiac or metabolic conditions that predisposed to AF. The AF resolved within three hours of onset without intervention.

**Conclusion:**

Cannabis use should be considered as a possible etiology of new-onset AF, especially in relatively young patients with no other predisposing risk factors.

CPC-EM Capsule What do we already know about this clinical entity?

*Atrial fibrillation (AF) occurs primarily in individuals with known risk factors. Marijuana use has several proarrhythmic effects on the heart.*

What is the major impact of the image(s)?

*A patient with no risk factors for AF presented to the ED with paroxysmal AF after smoking marijuana. No other cause of her AF was identified after hospital admission.*

How might this improve emergency medicine practice?

*Use of cannabis should be considered as a possible etiology of new-onset AF, especially in patients with no other predisposing factors.*

## INTRODUCTION

Atrial fibrillation (AF) is the most common cardiac arrhythmia, occurring in 1–3% of the general population and in up to 17% of individuals >80 years in age.^1^ The majority of AF cases occur in patients with known risk factors such as advanced age, heart failure, and coronary artery disease.^1^ We report a case of paroxysmal AF in a 38-year-old woman with no associated risk factors after smoking marijuana.

## CASE REPORT

A 38-year-old woman with a history of obesity presented to the emergency department (ED) complaining of palpitations with associated shortness of breath, dizziness, and chest discomfort. The symptoms had begun abruptly approximately one hour earlier while she was a patient in a nearby dental clinic. She admitted to recreational marijuana use and reported she had smoked marijuana just prior to her dental appointment to alleviate her related anxiety.

On physical examination, she had a pulse of 93 beats per minute with an irregular rhythm, and her blood pressure was 123/90 millimeters of mercury. Her lungs were clear to auscultation. An electrocardiogram revealed an irregular rhythm with a narrow QRS complex and an absence of P waves, consistent with AF (Image). No ischemic changes were present.

**Image.**
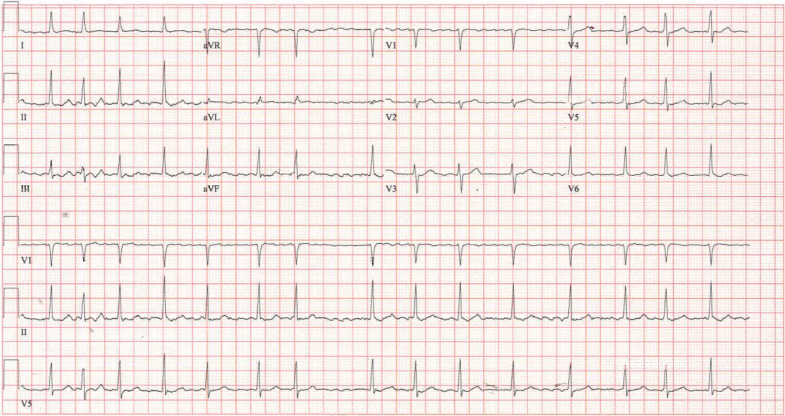
The patient’s electrocardiogram showing atrial fibrillation.

Laboratory testing in the ED was remarkable for a minimally elevated serum high-sensitivity troponin I level of 15 nanograms per liter (ng/L) (reference range: 0–15 ng/L) and a serum thyroid stimulating hormone level in the normal range. Approximately 90 minutes after ED arrival, the patient spontaneously converted to a normal sinus rhythm. She was admitted to the hospital for evaluation of new-onset AF.

The patient remained in a sinus rhythm while in the hospital. Serial serum high-sensitivity troponin I levels were not indicative of myocardial damage. Her serum magnesium level was found to be just below the normal range at 1.7 milligrams per deciliter (mg/dL) (1.8–2.4 mg/dL). Her urine toxicological screening was positive only for cannabinoids. Her transthoracic echo revealed no structural abnormalities of the heart and normal left ventricular function. The patient was discharged home on hospital day two on no new medications as her AF was felt to be paroxysmal in nature.

## DISCUSSION

Cannabis is the most widely used illicit drug in the United States (US).^2^ The percentage of US adults that use marijuana has more than doubled since 2001, and cannabis in various forms for recreational or medicinal use is now legal in 46 states.^3^ ^,^ ^4^ The potency of cannabis has also increased significantly in the past 20 years in the US.^5^ As of 2021, approximately 75% of US adults believed cannabis use was as safe or safer than tobacco use.^6^

Paroxysmal AF following marijuana use was first reported in 2000.^7^ Since then, several similar cases have been described, often in young people with structurally normal hearts and no other risk factors for AF.^8^ It is possible that AF after marijuana use is under-reported because patients are reluctant to seek medical care while under the influence of the drug due to legal concerns. Also, palpitations suggestive of AF experienced by users may be interpreted as simply an expected cardiovascular effect of cannabis use. Atrial fibrillation following use of synthetic cannabinoids has also been described.^9^

Various cardiac arrhythmias have been reported with marijuana use, with AF and ventricular fibrillation the most common (Table).^10^ ^–^ ^12^ The mechanisms by which marijuana induces cardiac arrhythmias is unknown, although several mechanisms have been proposed (Figure).^8^ ^–^ ^10^ Autonomic dysfunction begins within minutes after smoking marijuana, causing a biphasic and dose-dependent effect. Sympathetic stimulation is predominant at lower doses, leading to tachycardia and enhanced cardiac automaticity. At higher doses, parasympathetic activation leads to bradycardia and hypotension.^8^ ^,^ ^13^ Cannabis may also induce coronary vasospasm and have a detrimental effect on the coronary microcirculation, leading to myocardial ischemia.^8^ ^,^ ^10^ All of these cardiovascular changes result in proarrhythmic effects on the heart.

**Table.** Cardiac arrhythmias reported with marijuana use.

Supraventricular arrhythmias	Ventricular arrhythmias
Atrial fibrillation	Ventricular tachycardia
Atrial flutter	Ventricular fibrillation
Ectopic atrial tachycardia	Asystole
Supraventricular tachycardia	
Junctional rhythm	
Sinus bradycardia	
Sinus arrest	
First degree AV block	
Second degree AV block	
Third degree AV block	

*AV*, atrioventricular.

**Figure.**
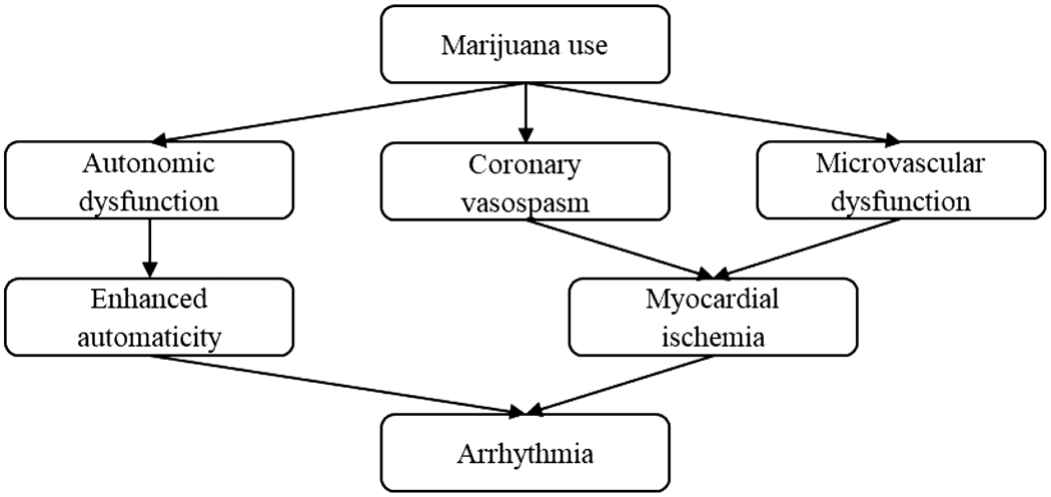
Proposed mechanisms of marijuana-induced cardiac arrhythmias.

The incidence of AF related to marijuana use is difficult to estimate, although the prevalence of cannabis use disorder among patients admitted for AF is increasing, with the greatest increase in percentage prevalence among younger patients.^14^ Atrial fibrillation related to marijuana use at this point seems to be a transient and relatively benign phenomena, leading some authors to compare it to “holiday heart” occurring with ethanol use.^15^ Screening for cannabis use by history or laboratory testing should be considered in patients with new-onset AF, especially in those otherwise young and healthy.

## CONCLUSION

Atrial fibrillation associated with marijuana use has been increasingly described in the past 20 years. The exact mechanism by which marijuana induces AF is unknown. This phenomenon is typically transient and relatively benign in patients that are otherwise young and healthy. Cannabis use should be considered as a possible etiology of new onset AF, especially in young patients with no other predisposing risk factors.
